# Adherence to Clotting Factor Prophylaxis in Adolescent and Adult Males With Haemophilia

**DOI:** 10.1111/hae.70244

**Published:** 2026-03-23

**Authors:** Nathália Martins Beserra, Ricardo Mesquita Camelo, Alice Oliver Rosa Sacramento, Eliziane Souza Nascimento, Rosângela Pinheiro Gonçalves Machado, Clarissa Maria Gonçalves Machado, Suzzy Maria Carvalho Dantas, Samuel Gonçalves Machado da Rocha, Luany Elvira Mesquita Carvalho, Romélia Pinheiro Gonçalves Lemes

**Affiliations:** ^1^ Centro de Hematologia e Hemoterapia do Ceará (HEMOCE) Fortaleza Brazil; ^2^ Faculty of Medicine Universidade Federal de Minas Gerais (UFMG) Belo Horizonte Brazil; ^3^ Fundação Hemominas Belo Horizonte Brazil; ^4^ Universidade Federal do Ceará (UFC) Fortaleza Brazil

**Keywords:** adherence, clotting factor, haemophilia, prophylaxis, quality of life

## Abstract

**Introduction:**

Clotting factor prophylaxis remains the most prescribed standard‐of‐care treatment for people with haemophilia (PwH). Prophylaxis prevents bleeds, joint damage, and improves quality of life (QoL). Its success depends on treatment adherence, consisting of socioeconomic, behavioural, and disease‐ or therapy‐related factors.

**Aim:**

To identify determinants of clotting factor prophylaxis adherence among PwH followed at a Hemophilia Treatment Center (HTC) in Brazil.

**Methods:**

A cross‐sectional study was conducted at a Brazilian HTC in June–October/2022. Eligible participants were PwH A or B without inhibitors and on clotting factor prophylaxis, aged ≥ 14 years. Demographic, clinical, and therapeutic data were collected from records and interviews. Health‐related QoL was assessed by the SF‐36 questionnaire. Adherence was assessed using the Brazilian validated Validated Hemophilia Regimen Treatment Adherence Scale‐Prophylaxis (VERITAS‐Pro) questionnaire.

**Results:**

Among 78 PwH (median age 30 years), 70 (90%) had severe haemophilia, 46 (59%) reported joint impairment, and 67 (86%) infused 3 times/week. Median SF‐36 score was 70.3 (interquartile range [IQR] 57.3–83.3). Median VERITAS‐Pro score was 44.0 (IQR 35.0–50.0), resulting in 70 (90%) adherent PwH. SF‐36 score was not correlated with VERITAS‐Pro. Clotting factor prophylaxis adherence was associated with having haemophilia A (*p* = 0.012) and infusing factor >2 times weekly (*p* = 0.008), and inversely correlated with prophylaxis duration (*r* = 0.317 [weak clinical correlation]; *p* = 0.005), and directly correlated with SF‐36 domain “Mental health” (*r* = −0.304 [weak clinical correlation]; *p* = 0.007).

**Conclusion:**

The main factors determining adherence to clotting factor prophylaxis were having haemophilia A, receiving factor infusions more than twice weekly, having a shorter duration of prophylaxis, and good mental health.

## 1 Introduction

Hereditary haemophilia is a rare X‐linked bleeding disorder characterised by reduced or absent plasma activity of clotting factors VIII (FVIII), in haemophilia A (HA), and IX (FIX), in haemophilia B (HB) [1]. Clinically, haemophilia presents with spontaneous bleeding or bleeding triggered by minor trauma [2]. Joint bleeding (hemarthrosis) is common and may result in arthropathy, chronic pain, functional impairment, and reduced quality of life (QoL) [3, 4].

Prophylaxis is currently the gold standard for preventing bleeding episodes in PwH, consisting of regular administration of procoagulant therapies to maintain haemostatic protection [2]. For several decades, prophylaxis has been predominantly based on regular intravenous infusions of the deficient clotting factor (FVIII or FIX), which can prevent bleeds, joint damage, and improve QoL. More recently, non‐factor therapies such as emicizumab for PwHA and concizumab for both PwHA and PwHB have expanded prophylactic options [5, 6]. However, prophylaxis with emicizumab or concizumab is not yet globally adopted for PwH without neutralizing antibodies against clotting factors (i.e., inhibitors), and clotting factor prophylaxis remains the most common approach in settings where prophylaxis is standardised [7].

Frequent intravenous administration of clotting factors as prophylaxis maintains a higher residual plasma activity, thereby ensuring haemostasis [2]. As a result, fewer haemarthroses occur and, consequently, less joint damage [3, 8]. In the long term, this leads to musculoskeletal improvements along with better QoL [3, 8]. Regarding non‐factor products, the benefits related to bleeding reduction are still under evaluation [9].

Regardless of context, the success of prophylaxis is multifactorial, involving socioeconomic determinants, behaviours and beliefs, and disease‐ and therapy‐related characteristics [8, 10]. Given the significant impact of prophylaxis on multiple disease outcomes, treatment adherence is a crucial aspect [8]. A recent systematic review showed that higher adherence is associated with symptom experience, positive patient‐provider relationships, and a strong belief in the need for treatment, whereas lower adherence is linked to the absent or infrequent symptoms and increasing age [11].

Therefore, identifying the determinants of clotting factor prophylaxis adherence is essential for developing individualised interventions by the interdisciplinary team to optimise outcomes [12]. Accordingly, this study aimed to identify the determinants of clotting factor prophylaxis adherence among PwH followed at a Hemophilia Treatment Center (HTC) in Brazil.

## 2 Methods

### 2.1 General Information

A cross‐sectional study was conducted at the Coagulation Disorders Outpatient Clinic of Centro de Hematologia e Hemoterapia do Ceará (HEMOCE), in Fortaleza/Brazil, in June–October/2022. The study was approved by the HEMOCE Committee on Ethics on Research (CAAE 56638022.6.0000.8152, 04/28/2022). All participants signed a consent form to use personal data accumulated in the database. For PwH under 18 years old, their legal guardians answered the questionnaires together with the PwH.

### 2.2 Study Population

Inclusion criteria were PwH on clotting factor prophylaxis with FVIII or FIX, negative inhibitor titre, and registration in the Hemovida WebCoagulopathies System with regular follow‐up at HEMOCE‐Fortaleza [13]. For the current analysis, only PwH aged 14 years or older were evaluated, according to the eligibility requirements of the Short Form 36 (SF‐36) QoL questionnaire [14]. PwH who were bedridden or incarcerated were excluded. All PwH meeting the inclusion criteria were consecutively invited to participate in the study during routine visits.

At the time of the study, the Brazilian Ministry of Health was responsible for the purchase and distribution of clotting factors for the treatment of haemophilia with no additional cost to patients [13]. Treatment access was dependent on registration in a national database [13], and treatment followed guidelines nationally established [15]. For PwHA, recombinant FVIII (for those under 40 years) or plasma‐derived FVIII (for those over 40 years) was available. For PwHB, plasma‐derived FIX was provided. Integrated haemophilia support was provided for free at Brazilian HTC, consisting of an interdisciplinary team.

Clotting factor prophylaxis was defined as the regular and continuous clotting factor prescription aimed at preventing bleeding, covering at least 45 weeks per year [2, 15].Primary prophylaxis was initiated in the absence of a documented joint disease, before the second joint bleed and before 3 years old. Secondary prophylaxis was initiated after two or more joint bleeds or after 3 years old, but before joint disease was detected. Tertiary prophylaxis was initiated after the identification of documented joint disease, regardless of the number of bleeding episodes or age [2, 15]. Inhibitor titres were tested at least yearly [15]. A positive inhibitor was defined as a titre ≥ 0.6 BU/mL, in two separate tests at least two weeks apart, using the Nijmegen–Bethesda assay [2, 15].

### 2.3 General Characteristics

The evaluated characteristics were selected from a literature review and improved with affordable data from the HTC reality (NMB, RMC, and RPGL). A structured form was developed by the lead researcher (NMB), which included the following sections: sociodemographic data of the PwH and their caregiver, clinical profile data, and treatment‐related data. Data were collected from medical records and prescriptions, and interviews.

Sociodemographic data of the PwH and their caregiver included age at study entrance, sex, region of residence, educational level, self‐reported skin colour, marital status, and occupation [16]. For caregivers, the degree of kinship to the PwH, responsibility for administering infusions, and whether they received training at the HTC were also recorded.

Clinical profile data included haemophilia type and severity [2], serological status for human immunodeficiency virus (HIV) and hepatitis C virus (HCV) [15], joint impairment [4], presence of other diseases, type and frequency of physical activity, treatment of haemarthroses at the HTC, and family history of haemophilia. Haemophilia severity was defined by the lowest recorded plasma factor activity at diagnosis, in the absence of replacement therapy in the preceding 48 h (HA) or 72 h (HB), and with a negative inhibitor titre [2]. Plasma factor activity defined mild, moderate, and severe haemophilia as 5.1%–40%, 1%–5%, and < 1% of normal, respectively, based on Nijmegen–Bethesda assay [17]. Joint impairment was defined as the presence of arthropathy in 2021, as recorded in the medical chart based on clinical assessment, physical examination, or imaging. Physical activity data were based on PwH self‐reports in the clinical profile questionnaire. The annual rate of haemarthroses treated at the HTC was calculated from the number of treated joint bleeding episodes over a 12‐month period.

Treatment‐related data included the person responsible for infusing the clotting factor, the reason why the PwH did not self‐infuse clotting factor when applicable, training received at the HTC for self‐infusion, age at prophylaxis start, type of prophylaxis [2], type of clotting factor, prophylaxis regimen (dose per infusion and weekly frequency), use of an infusion logbook [18], and history of immune tolerance induction [2]. Self‐infusion denotes the independent intravenous administration of clotting factor by the PwH themselves, performed outside the healthcare setting and without direct professional assistance at the time of infusion [2]. Infusion training refers to the structured person‐directed education and supervised skill acquisition provided by the HTC interdisciplinary team to enable safe and effective administration of clotting factor concentrates [2].

### 2.4 Quality of Life Assessment

We defined QoL as a multidimensional phenomenon encompassing physical, psychological, and social functioning, and can be assessed using standardised instruments [19]. The health‐related non‐hemophilia‐specific SF‐36 questionnaire, translated and validated in Brazil was applied to assess QoL among PwH aged over 14 years [14]. The SF‐36 is a multidimensional instrument composed of 36 items covering eight domains: General health, Social functioning, Mental health, Vitality, Bodily pain, Physical functioning, Role limitations (physical), and Role limitations (emotional) [14]. Each component is scored on a scale from 0 to 100, where 0 represents the worst and 100 represents the best health status [14].

### 2.5 Adherence to Prophylaxis Assessment

The Validated Hemophilia Regimen Treatment Adherence Scale‐Prophylaxis (VERITAS‐Pro), translated and validated in Brazil according to age group, was used to assess treatment adherence [20]. The questionnaire consists of six domains (Routine, Dosage, Planning, Remembering, Skipping, and Communicating), with four statements per domain. Respondents indicate the frequency with which each statement applies, ranging from ‘always’ to ‘never’. Depending on the direction of the statement, responses are scored on a Likert scale ranging from 1 to 5 or 5 to 1. Each domain yields a score ranging from 4 to 20, and the total score ranges from 24 to 120. Lower scores indicate better adherence. Adherence was defined as scores below 11 (Routine), 7 (Dosage), 9 (Planning), 11 (Remembering), 11 (Skipping), 10 (Communicating), and 57 (total score) [21].

### 2.6 Statistical Analysis

Data were collected and organised anonymously in a database created in Microsoft Excel 2010 (Microsoft Corporation, Redmond, US). Statistical analyses were performed using SPSS software, version 22 (IBM Corp., Armonk, US). There were no missing data for the comparative analyses.

To facilitate evaluation of treatment adherence (a continuous variable) according to the categorical variables, two consecutive steps were taken: firstly, categorical variables with more than two options were converted into binary variables; in sequence, only categorical variables with ≥ 4 individuals per option in the binary model were analysed.

Continuous variables were described using median, interquartile range (IQR), and range (minimum and maximum). Differences between medians were assessed using Mann–Whitney *U* test. The linear correlation between continuous variables was estimated using Pearson's *r* test. The clinical significance of the correlations was defined as very weak, if |*r*| < 0.200; weak, if |*r*| = 0.200–0.399; moderate, if |*r*| = 0.400–0.599; strong, if |*r*| = 0.600–0.799; and very strong, if |*r*| ≥ 0.800. Categorical variables were expressed as absolute numbers and relative frequencies (percentages). Differences between frequencies were assessed using Fisher's exact test.

## 3 Results

### 3.1 General Characteristics

A total of 78 PwH was included in the study (Figure S1), with 30.0 years (range 14–63) (Table 1). The questionnaires for the 7 (9%) PwH under 18 years were completed by their mothers, who were 36.0 years (range 32–42) (Table S1). All PwH were male (78; 100%), and 49 (63%) PwH were single (Table 1). Most PwH had HA (67; 86%) and severe disease (70; 90%). Joint impairment was reported by 46 (59%) PwH, and 7 (15%) of them had more than two joints affected. Haemarthrosis treatment at the HTC was recorded for 37 (47%) PwH, with an annual joint bleeding rate of 0.5 (range 0‐19). Half of the PwH (39; 50%) engaged in physical activity, mainly weight training (16; 41%). The main reasons for not performing self‐infusion in 17 (22%) PwH were lack of interest (8; 47%) and fear (8; 47%). In these cases, mothers were reported as responsible for infusions in 9 (53%) PwH. Prophylaxis duration was 9.0 years (range 1–12), with most PwH receiving secondary or tertiary prophylaxis (76; 97%). SF‐36 score was 70.3 (range 25.6–96.3) (Table 2).

**TABLE 1:** Population characteristics.

Characteristic	Result (*n* = 78)
**Sociodemographic data**	
Age, in years, as median (IQR), range	30.0 (20.8–39.0), 14–63
Teenager, in *n* (%)	7 (9%)
Male, in *n* (%)	78 (100%)
Region of residence, in *n* (%)	
Fortaleza	39 (50%)
Fortaleza Metropolitan Area	12 (15%)
Interior	27 (35%)
Educational level, in *n* (%)	
Illiterate	2 (3%)
Primary	20 (26%)
Secondary	21 (27%)
Higher education	30 (38%)
Postgraduate	5 (6%)
Self‐reported skin colour, in *n* (%)	
White	17 (22%)
Brown	47 (60%)
Black	14 (18%)
Marital status, in *n* (%)	
Single	49 (63%)
Married	26 (33%)
Separated/divorced	3 (4%)
Occupation, in *n* (%)	
Student	26 (33%)
Self‐employed	15 (19%)
Employed	13 (17%)
Public servant	3 (4%)
Retired due to disability	6 (8%)
Retired by length service	2 (3%)
Unemployed	20 (26%)
**Clinical data**	
Haemophilia type, in *n* (%)	
A	67 (86%)
B	11 (14%)
Haemophilia severity, in *n* (%)	
Severe	70 (90%)
Moderate	7 (9%)
Mild	1 (1%)
HIV infection, in *n* (%)	1 (1%)
History of HCV infection, in *n* (%)	7 (9%)
Joint impairment, in *n* (%)	46 (59%)^a^
Other disease, in *n* (%)	16 (21%)^b^
Physical activity practice, in *n* (%)	39 (50%)^c^
Treatment of haemarthroses at the HTC, in *n* (%)	37 (47%)^d^
Family history of haemophilia, in *n* (%)	48 (61%)
**Treatment data**	
Self‐infusion, in *n* (%)	61 (78%)^e^
Infusion training, in *n* (%)	40 (51%)
Duration of prophylaxis, in years, in median (IQR), range	9.0 (7.0–10.0), 1–12
Type of prophylaxis, in *n* (%)	
Primary prophylaxis	2 (3%)
Secondary/tertiary prophylaxis	76 (97%)
Dose per infusion, in *n* (%)	
< 20 IU/kg	3 (4%)
20–30 IU/kg	47 (60%)
> 30 IU/kg	28 (36%)
Infusion frequency, in *n* (%)	
2x/week	10 (13%)
3x/week	67 (86%)
Every other day	1 (1%)
Infusion diary, in *n* (%)	4 (6%)
Previous immune tolerance induction, in *n* (%)	6 (8%)

Abbreviations: HCV, hepatitis C virus; HIV, human immunodeficiency virus; HTC, Hemophilia Treatment Center; IQR, interquartile range; PwH, people with haemophilia.

^a^ Of the PwH with arthropathy, 19 (41%) had 1 affected joint, 20 (44%) had 2 affected joints, and 7 (15%) had more than 2 affected joints.

^b^ Of the PwH who reported having other diseases, 1 had asthma, 1 had heart disease, 1 had type 1 diabetes mellitus, 1 had HIV, 1 had sleep apnea, 2 had type 2 diabetes mellitus, 3 had chronic kidney disease, 5 had systemic arterial hypertension, and 7 had HCV.

^c^ The reported physical activities were capoeira (*n* = 1), physical education (*n* = 1), volleyball (*n* = 1), surfing (*n* = 1), swimming (*n* = 1), cycling (*n* = 7), soccer (*n* = 4), walking (*n* = 10), and free exercises (*n* = 16).

^d^ Of the PwH with haemarthrosis treated at the HTC, 19 (51%) had 1 event, 9 (19%) had 2 events, and 11 (30%) had more than 2 events. The annual bleeding rate was 0.5 bleed (IQR 0.0–2.0), range 0–19 bleeds.

^e^ The reasons not to perform self‐infusion were being a child (*n* = 1), having physical disability (*n* = 1), having visual disability (*n* = 1), lacking interest (*n* = 8), and having fear (*n* = 8). Those who performed the infusion in these PwH were wives (*n* = 3), mothers (*n* = 9), sisters (*n* = 2), friends (*n* = 2), professional at the HTC (*n* = 3), professional at the hospital (*n* = 1), and professional at outpatient clinics (*n* = 4).

**TABLE 2:** Quality of life.

Characteristic	Results (*n* = 78)
General health, as median (IQR), range	62.0 (42.0–72.0), 5–100
Social functioning, as median (IQR), range	75.0 (59.5–100.0), 0–100
Mental health, as median (IQR), range	92.0 (72.0–100.0), 28–100
Vitality, as median (IQR), range	75.0 (55.0–85.0), 15–100
Bodily pain, as median (IQR), range	62.0 (41.0–90.0), 10–90
Physical functioning, as median (IQR), range	72.5 (40.0–95.0), 10–100
Role limitations (physical), as median (IQR), range	100.0 (25.0–100.0), 0–100
Role limitations (emotional), as median (IQR), range	100.0 (33.0–100.0), 10–100
Total SF‐36, as median (IQR), range	70.3 (57.3–83.3), 25.6–96.3

Abbreviations: IQR, interquartile range; SF‐36, Short Form 36.

### 3.2 Prophylaxis Adherence (VERITAS‐Pro)

Total VERITAS‐Pro score was 44.0 (range 24–67), with 70 (90%) PwH classified as adherent (Table 3). The domain with the lowest score (indicating better adherence) was Dose (4.0; range 4–11), with 66 (85%) adherent PwH, while the highest score was in the Communicate domain (9.0; range 4–20), with only 41 (53%) adherent PwH. However, the highest adherence rate was observed in the Remember domain (73; 94%).

**TABLE 3:** Adherence to clotting factor prophylaxis.

Characteristic	Score, as median (IQR), range (*n* = 78)	Adherent, in n (%) (*n* = 78)
Time	7.0 (5.0–9.0), 0–15	63 (81%)
Dose	4.0 (4.0–5.3), 4–11	66 (85%)
Plan	8.0 (8.0–10.0), 4–16	43 (55%)
Remember	5.0 (4.0–7.0), 4–13	73 (94%)
Skip	5.0 (4.0–7.0), 4–16	71 (91%)
Communicate	9.0 (5.8–13.3), 4–20	41 (53%)
Total VERITAS‐Pro	44.0 (35.0–50.0), 24–67	70 (90%)

Abbreviations: IQR, interquartile range; VERITAS‐Pro, Validated Hemophilia Regimen Treatment Adherence Scale‐Prophylaxis.

### 3.3 Total VERITAS‐Pro Score

Total VERITAS‐Pro adherence was associated with HA (*p* = 0.012) and infusing clotting factor more than twice weekly (*p* = 0.008), but not with QoL (Table 4). Total VERITAS‐Pro scores were lower (better adherence) among non‐white PwH compared to white PwH (*p* = 0.034), in PwHA compared to PwHB (*p* = 0.005), in users of infusion diaries versus non‐users (*p* = 0.001), in PwH who infused more than twice weekly compared to PwH who infused twice weekly (*p* = 0.003), in PwH with shorter duration of prophylaxis (*r* = 0.317 with a weak clinical correlation; *p* = 0.005), and in PwH with higher Mental health scores (*r* = −0.304 with a weak clinical correlation; *p* = 0.007) (Table S2). Analysis per domain scores were described in the Supplementary material.

**TABLE 4:** Population characteristics according to adherence to clotting factor prophylaxis (total VERITAS‐Pro).

Characteristic	Adherent (*n* = 70)	Nonadherent (*n* = 8)	p
Age, in years, in median (IQR)	30.0 (20.8–39.3)	25.0 (18.8–37.5)	0.604^*^
White, in *n* (%)	15 (21%)	2 (25%)	1.000^*^
Single, in *n* (%)	43 (61%)	6 (75%)	0.703^*^
Literate, in *n* (%)	68 (97%)	8 (100%)	1.000^*^
Living in Fortaleza, in *n* (%)	36 (51%)	3 (38%)	0.711^*^
Haemophilia A, in *n* (%)	63 (90%)	4 (50%)	0.012^*^
Severe haemophilia, in *n* (%)	62 (89%)	8 (100%)	0.591^*^
Positive joint impairment, in *n* (%)	41 (59%)	5 (63%)	1.000^*^
Positive other disease, in *n* (%)	11 (16%)	0 (0%)	0.592^*^
Positive physical activity practice, in *n* (%)	33 (47%)	6 (75%)	0.263^*^
Haemarthroses treated at the HTC, in *n* (%)	34 (49%)	3 (38%)	0.715^*^
Infusion training, in *n* (%)	38 (54%)	2 (25%)	0.149^*^
Self‐infusion, in *n* (%)	55 (79%)	6 (75%)	1.000^*^
Infusion diary, in *n* (%)	4 (6%)	0 (0%)	1.000^*^
Dose ≤ 30 IU/kg, in *n* (%)	26 (37%)	2 (25%)	0.704^*^
Frequency > 2x/week, in *n* (%)	64 (91%)	4 (50%)	0.008^*^
Duration of prophylaxis, in years, in median (IQR)	9.0 (7.0–10.0)	8.5 (8.0–11.8)	0.338^†^
General health, in median (IQR)	61.0 (42.0–72.0)	69.5 (56.8–79.3)	0.106^†^
Social functioning, in median (IQR)	75.0 (62.5–100.0)	66.8 (50.0–96.9)	0.750^†^
Mental health, in median (IQR)	92.0 (76.0–100.0)	76.0 (72.0–89.0)	0.109^†^
Vitality, in median (IQR)	75.0 (55.0–85.0)	60.0 (55.0–78.8)	0.349^†^
Bodily pain, in median (IQR)	61.5 (41.0–84.0)	87.0 (50.3–90.0)	0.206^†^
Physical functioning, in median (IQR)	72.5 (40.0–95.0)	80.0 (57.5–100.0)	0.236^†^
Role limitations (physical), in median (IQR)	100.0 (25.0–100.0)	87.5 (12.5–100.0)	0.986^†^
Role limitations (emotional), in median (IQR)	100.0 (33.0–100.0)	100.0 (8.3–100.0)	1.000^†^
Total SF‐36, in median (IQR)	69.3 (56.1–83.3)	65.6 (73.2–82.2)	0.615^†^

Abbreviations: HTC, Hemophilia Treatment Center; IQR, interquartile range; SF‐36, Short Form 36; VERITAS‐Pro, Validated Hemophilia Regimen Treatment Adherence Scale‐Prophylaxis.

* Fisher's exact test.

^†^ Mann‐Whitney's *U* test.

## 4 Discussion

The current study demonstrated a high clotting factor prophylaxis adherence rate among PwH treated at a Brazilian HTC. Having HA and infusing clotting factor more than twice weekly were associated with better prophylaxis adherence. However, no association was found between prophylaxis adherence and QoL.

According to the World Health Organization, adherence to treatment for chronic conditions is estimated at around 50% [22]. This contrasts with our clotting factor prophylaxis adherence rate of 90%. Other studies using the VERITAS‐Pro have reported adherence rates ranging from 56% to 94% [23, 24, 25, 26]. However, adherence varied by domain. In our study, the highest and the lowest adherence rates were observed in the Remember and the Communicate domains, respectively. This differs from another Brazilian study, which reported the highest and the lowest adherence rates in the Communicate and the Time domains, respectively [20]. In a Polish study, the highest and the lowest adherence rates were in the Dose and the Remember domains, respectively [26], while studies in Germany and China identified the highest adherence rate in the Plan domain and the lowest adherence rate in the Communicate domain [27, 28]. The Chinese authors suggested the use of digital technologies to improve communication between PwH and HTC [28]. Tools such as electronic diaries and telehealth platforms have shown potential to support haemophilia self‐management by facilitating bleeding event monitoring and treatment adjustments [29, 30, 31]. These differences among studies can be explained by differences in healthcare systems, patient populations, and cultural contexts, as well as variations in study design, sample size, and interpretation of the adherence [32]. In contrast, a Nigerian study found that fewer weekly infusions were associated with higher adherence [24]. These discrepancies reinforce that treatment adherence is a complex, multifactorial behaviour. As such, these differences may be influenced by many factors, such as, but not solely, disease severity, availability of treatment products, and perceptions of PwH about the importance of their treatment. Furthermore, local healthcare practices and the degree of family or community support may shape adherence patterns in different contexts.

We observed high QoL scores, with no association between QoL and clotting factor prophylaxis adherence. This aligns with findings from a Danish study using hemophilia‐specific QoL instruments [33], but not with another study [34], The lack of association in our study suggests that clotting factor prophylaxis adherence may be influenced by multiple factors beyond PwH perception of QoL. Other emotional, social, and cultural factors not evaluated by a QoL questionnaire can also play a critical role [35], This reinforces the importance of patient‐centred approaches that integrate broader dimensions of care, including active listening, psychosocial support, and attention to the lived experiences of PwH [25, 36].

Sociodemographic factors such as educational level, distance from the HTC, employment status, and having another family member with haemophilia were not associated with clotting factor prophylaxis adherence in our study. This may reflect the positive impact of interdisciplinary HTC team efforts, which support PwH through education, training, and encouragement of self‐management, thereby mitigating the impact of social inequalities [37]. In addition, the HTC is designed in a decentralised network, sharing care not only directly with PwH and their families, but also with local healthcare services such as community clinics and hospitals that equally provide support to them. These findings are consistent with the review by Schrijvers et al. [11], which highlighted the importance of relationships with the healthcare team and belief in the necessity of treatment as key adherence drivers [11]. Understanding PwH experiences is essential to identifying facilitators and barriers to adherence. This underscores the need for HTC teams to address not only clinical but also psychosocial aspects of care, given their direct impact on treatment engagement [25].

No association was found between adherence to prophylaxis and the presence of arthropathy, different from a previous study that showed that PwH with arthropathy may manage infusions more effectively, possibly due to the physical limitations, pain, and impact on daily activities [25]. A possible explanation is that many PwH in the study started prophylaxis already at high risk or with pre‐existing joint impairment. In Brazil, clotting factor prophylaxis was implemented as a public policy only in 2011 [13]. Therefore, many PwH aged ≥ 14 years in 2022 likely experienced haemarthroses prior to starting prophylaxis. Consequently, joint disease was similarly prevalent among adherent and non‐adherent participants.

Finally, we are aware that although clotting factor prophylaxis remains the standard of care in many parts of the world, the introduction of non‐factor therapies, such as emicizumab and concizumab, may help improve treatment adherence [38], due to lower dosing frequency and easier administration. Furthermore, gene therapy has shown promising results and may offer a potential one‐time treatment option for haemophilia in the future.

### 4.1 Limitations

The current study presents limitations. First, the high clotting factor prophylaxis adherence rate may have resulted in a ceiling effect. Therefore, many potential associations may not have been detected, reducing the statistical power of the analysis. The ceiling effect may have been influenced by multiple factors, including the sample size and the sensitivity of the instrument used to assess adherence. Regarding the study population, PwH were recruited from a single HTC with regular access to clotting factors, treatment modalities, and interdisciplinary care, which may have contributed to high adherence rates and, ultimately, limited the generalizability of the findings. As for the VERITAS‐Pro tool, we noted that high adherence rates have also been reported in other countries [23, 24, 25, 26], raising questions about the tool sensitivity [21]. VERITAS‐Pro was developed in the United States, where the healthcare system differs substantially from the Brazilian hemophilia‐care system [13, 39]. In Brazil, haemophilia interdisciplinary support and treatment are provided entirely free of charge to PwH, which may influence various adherence‐related determinants. Another important consideration is that VERITAS‐Pro is a self‐report instrument, which may lead to overestimation of adherence levels. There was no other instrument to measure clotting factor prophylaxis adherence specific for haemophilia, and we did not have access to non‐hemophilia‐specific tools. In addition, objective evaluation of prophylaxis adherence (e.g., medication dispensing or refill data, electronic monitoring systems) was not practical [40], and we could not rely on the infusion diary as a strategy to notify treatment due to its low rate of use.

Secondly, the concept of adherence can vary between patients and healthcare professionals, leading to differing interpretations [41]. For example, while clinicians often focus on measurable aspects such as how often and when treatments are administered, patients and their caregivers tend to evaluate adherence based on their personal experience of the treatment demands and the effort required to maintain it.

Thirdly, we were unable to assess the association between adherence to clotting factor prophylaxis and bleeding outcomes. Bleeding episodes were not systematically recorded through infusion diaries, as we showed a small percentage of PwH used them, and documentation in medical records was inconsistent, precluding reliable analysis. Although adherence is generally expected to reduce bleeding, prior studies have reported heterogeneous findings [42, 43, 44]. Consequently, evaluation of bleeding outcomes was not feasible in the present study.

Finally, it is important to recognise that other possible determinants may interfere with the regularity of clotting factor infusions and we may not have evaluated them. For instance, delays in providing or collecting clotting factor concentrates may not be fully captured by standardised tools that rely on patient self‐report.

## 5 Conclusion

The main determinants of clotting factor prophylaxis adherence among PwH aged ≥ 14 years treated at a Brazilian HTC were having haemophilia A, infusing factor more than twice weekly, having a shorter duration of prophylaxis, and a good mental health.

## Author Contributions

NMB was responsible for Data curation, Investigation, Writing – original draft, and Writing – review & editing. RMC was responsible for Conceptualization, Formal analysis, Methodology, Resources, Validation, Visualization, Writing – original draft, and Writing – review & editing. AORS was responsible Visualization, Writing – original draft, and Writing – review & editing. ESN was responsible for Writing – original draft, and Writing – review & editing. RPGM was responsible for Writing – review & editing. CMGM was responsible for Writing – review & editing. SMCD was responsible for Writing – review & editing. SGMR was responsible for Writing – review & editing. LEMC was responsible for Writing – review & editing. RPGL was responsible for Investigation, Methodology, Project administration, Supervision, Writing – original draft, and Writing – review & editing.

## Funding

The authors have nothing to report.

## Ethics Statement

This study was conducted in accordance with the principles of the Declaration of Helsinki. The research protocol was approved by the Research Ethics Committee of the Centro de Hematologia e Hemoterapia do Ceará (HEMOCE) (CAAE 56638022.6.0000.8152; 28, April 2022). All participants provided written informed consent prior to participation. Confidentiality and anonymity were ensured throughout the study.

## Conflicts of Interest

Nathália Martins Beserra received speaker fees from Roche, Novo Nordisk, Takeda, and Bayer; and grants for scientific events from Roche, Novo Nordisk, and Sanofi. Ricardo Mesquita Camelo received speaker fees from Bayer, Novartis, Roche, and Takeda; and grants for scientific events from Novartis, Bayer, Novo Nordisk, Roche, and Takeda. AORS received grants for scientific events from Roche and Takeda; and wrote scientific material from Sanofi. Luany Elvira Mesquita Carvalho received speaker fees from Roche and Novo Nordisk; and grants for scientific events from Roche, Novo Nordisk, Takeda, and Sanofi. Eliziane Souza Nascimento, Rosângela Pinheiro Gonçalves Machado, Clarissa Maria Gonçalves Machado, Suzzy Maria Carvalho Dantas, Samuel Gonçalves Machado da Rocha, and Romélia Pinheiro Gonçalves Lemes have no interests that may be perceived as posing a conflict or bias.

## Declaration of Use of Artificial Intelligence in Scientific Writing

During the preparation of this manuscript, the authors did not use artificial intelligence tools.

## Declaration of Use of Medical Writing

During the preparation of this manuscript, the authors did not seek the assistance of a medical writer.

## Previous Presentation of Partial Results

HEMO 2025 São Paulo

BIC 2025 Padova

ISTH 2024 Bangkok

Hemo 2023 São Paulo

## Supporting information




**Supporting File 1**: Hae70244‐sup‐0001‐SuppMat.Docx.

## Data Availability

Data available on request due to privacy/ethical restrictions.
