# Supplementary material for: Adherence to Clotting Factor Prophylaxis in Adolescent and Adult Males With Haemophilia
Source: Haemophilia. 2026 Mar 23;32(3):739–47. doi: 10.1111/hae.70244 (PMC13175437; doi:10.1111/hae.70244)
Supplement: Supplementary file 1 — Supporting File 1: Hae70244‐sup‐0001‐SuppMat.Docx. [file HAE-32-739-s001.docx]

**SUPPLEMENTARY MATERIAL**

**Adherence to clotting factor prophylaxis in adolescent and adult males with hemophilia**

Nathália Martins Beserra, Ricardo Mesquita Camelo, Alice Oliver Rosa Sacramento, Eliziane Souza Nascimento, Rosângela Pinheiro Gonçalves Machado, Clarissa Maria Gonçalves Machado, Suzzy Maria Carvalho Dantas, Samuel Gonçalves Machado da Rocha, Luany Elvira Mesquita Carvalho, Romélia Pinheiro Gonçalves Lemes

**Index**

Results, VERITAS-Pro Domain Scores ……………………………………………………………..… page 2

Supplementary Table 1 ......................................................................................... page 3

Supplementary Table 2 ......................................................................................... page 4

Supplementary Table 3 ......................................................................................... page 7

STROBE ……………………………………………………………………………………………………………. page 8

Supplementary Figure 1 …………………………………………………………………………………… page 10

**Results**

**VERITAS-Pro Domain Scores**

Adherence assessed by the Time domain was associated with non-white PwH (p = 0.016), HA (p = 0.005), receiving a dose ≤ 30 IU/kg (p = 0.039), and infusing more than twice weekly (p = 0.019) (Supplementary Table 3). Time domain scores were lower (better adherence) in PwHA compared to PwHB (p = 0.011), in PwH with other diseases compared to PwH without (p = 0.043), in users of infusion diaries (p = 0.012), and in PwH who infused more than twice weekly (p = 0.035) (Supplementary Table 2).

Adherence assessed by the Dose domain was associated with higher scores in General health (p = 0.028), Social functioning (p = 0.025), Vitality (p = 0.003), Bodily pain (p = 0.017), and the overall SF-36 score (p = 0.005) (Supplementary Table 3). Dose domain scores were lower (better adherence) in PwH with higher scores in General health (*r* = –0.252; p = 0.026), Social functioning (*r* = –0.297; p = 0.008), Mental health (*r* = –0.260; p = 0.021), Vitality (*r* = –0.395; p < 0.001), Bodily pain (*r* = –0.286; p = 0.011), and SF-36 (*r* = –0.302; p = 0.007) (Supplementary Table 2).

Adherence assessed by the Plan domain was associated with higher Mental health (p = 0.027) and Vitality (p = 0.018) scores (Supplementary Table 3). Plan domain scores were lower (better adherence) in infusion diary users (p = 0.018) and PwH with higher Mental health scores (*r* = –0.285; p = 0.011) (Supplementary Table 2).

Adherence assessed by the Remember domain was associated with HA (p = 0.001) (Supplementary Table 3).

Adherence assessed by the Skip domain was associated with HA (p = 0.006), absence of arthropathy (p = 0.017), and infusing more than twice weekly (p = 0.004) (Supplementary Table 3). Skip domain scores were lower (better adherence) in PwHA compared to PwHB (p = 0.025), in infusion diary users (p = 0.017), in PwH who infused more than twice weekly (p = 0.006), and in PwH with shorter prophylaxis duration (*r* = 0.251; p = 0.027) (Supplementary Table 2).

Adherence assessed by the Communicate domain was associated with not engaging in physical activity (p = 0.023) and shorter duration of prophylaxis (p = 0.030) (Supplementary Table 3). Communicate domain scores were lower (better adherence) in infusion diary users (p = 0.042) and in PwH with shorter prophylaxis duration (*r* = 0.326; p = 0.004) (Supplementary Table 2).

**Supplementary Table 1- Guardians characteristics**

| **Characteristic** | **Result (n = 7)** |
| --- | --- |
| Mother, in n (%) | 7 (100%) |
| Age, in years, as median (IQR), range | 36.0 (36.0-41.0), 32-42 |
| Self-reported skin color, in n (%)  Brown  Black | 6 (86%)  1 (14%) |
| Educational level, in n (%)  Fundamental complete  Medium complete  Superior complete | 1 (14%)  4 (57%)  2 (29%) |

IQR: interquartile range.

**Supplementary Table 2- Adherence to clotting factor prophylaxis scores (VERITAS-Pro total and domains) according to population characteristics**

| **Characteristic** | **n** | **VERITAS-Pro** | | | | | | |
| --- | --- | --- | --- | --- | --- | --- | --- | --- |
|  |  | **Time** | **Dose** | **Plan** | **Remember** | **Skip** | **Communicate** | **Total** |
| Age  < 18 years  ≥ 18 years  p* | 7  71 | 6.0 (5.0-8.0)  7.0 (5.0-9.0)  0.335 | 4.0 (4.0-5.0)  4.0 (4.0-6.0)  0.547 | 8.0 (8.0-10.0)  8.0 (8.0-10.0)  0.978 | 4.0 (4.0-6.0)  5.0 (4.0-7.0)  0.585 | 4.0 (4.0-6.0)  6.0 (4.0-7.0)  0.266 | 8.0 (7.0-10.0)  9.0 (5.0-14.0)  0.480 | 37.0 (34.0-44.0)  45.0 (35.0-50.0)  0.306 |
| Age  *r*  p^†^ | 78 | -0.058  0.613 | 0.107  0.352 | -0.072  0.530 | -0.177  0.122 | -0.052  0.649 | 0.021  0.855 | -0.057  0.618 |
| Residence municipality  Fortaleza  Interior  p* | 39  39 | 8.0 (6.0-9.0)  6.0 (5.0-9.0)  0.117 | 4.0 (4.0-5.0)  4.0 (4.0-6.0)  0.216 | 8.0 (8.0-11.0)  8.0 (8.0-10.0)  0.945 | 4.0 (4.0-6.0)  6.0 (4.0-7.0)  0.249 | 5.0 (4.0-7.0)  6.0 (4.0-8.0)  0.569 | 9.0 (5.0-14.0)  9.0 (7.0-13.0)  0.864 | 44.0 (36.0-50.0)  45.0 (33.0-50.0)  0.908 |
| Self- reported skin color  White  Other  p* | 17  61 | 9.0 (7.0-12.5)  6.0 (5.0-9.0)  0.003 | 4.0 (4.0-5.5)  4.0 (4.0-5.5)  0.573 | 8.0 (8.0-11.0)  8.0 (8.0-11.0)  0.783 | 6.0 (4.0-8.0)  5.0 (4.0-7.0)  0.282 | 7.0 (4.5-9.0)  5.0 (4.0-7.0)  0.081 | 10.0 (8.0-15.0)  9.0 (4.0-13.0)  0.222 | 48.0 (40.5-53.5)  44.0 (33.0-48.0)  0.034 |
| Marital status  Single  Other  p* | 49  29 | 7.0 (5.5-9.0)  7.0 (4.5-9.0)  0.765 | 4.0 (4.0-5.0)  4.0 (4.0-6.0)  0.600 | 8.0 (8.0-10.5)  8.0 (8.0-10.0)  0.397 | 6.0 (4.0-7.0)  4.0 (4.0-6.5)  0.155 | 6.0 (4.0-7.5)  5.0 (4.0-7.0)  0.457 | 9.0 (6.5-13.5)  11.0 (4.5-13.5)  0.909 | 46.0 (35.0-50.0)  43.0 (33.0-51.0)  0.441 |
| Hemophilia type  A  B  p* | 67  11 | 6.0 (5.0-9.0)  11.0 (6.0-13.0)  0.011 | 4.0 (4.0-5.0)  5.0 (4.0-6.0)  0.173 | 8.0 (8.0-10.0)  10.0 (8.0-11.0)  0.313 | 5.0 (4.0-7.0)  6.0 (4.0-12.0)  0.291 | 5.0 (4.0-7.0)  7.0 (5.0-11.0)  0.025 | 9.0 (4.0-14.0)  11.0 (8.0-12.0)  0.365 | 43.0 (34.0-48.0)  52.0 (45.0-58.0)  0.005 |
| Hemophilia severity  Severe  Other  p* | 70  8 | 6.5 (5.0-9.0)  7.5 (6.0-11.0)  0.438 | 4.0 (4.0-6.0)  4.0 (4.0-4.0)  0.047 | 8.0 (8.0-10.0)  8.5 (8.0-10.0)  0.924 | 5.0 (4.0-7.0)  7.0 (4.5-9.5)  0.066 | 5.0 (4.0-7.0)  5.5 (4.3-9.5)  0.600 | 9.5 (4.8-14.0)  9.0 (7.3-11.3)  0.842 | 44.0 (34.8-50.0)  44.0 (39.0-51.5)  0.798 |
| Joint disease  Yes  No  p* | 46  32 | 6.0 (5.0-9.0)  7.0 (6.0-9.0)  0.339 | 4.0 (4.0-6.0)  4.0 (4.0-5.0)  0.581 | 8.0 (8.0-10.0)  8.5 (8.0-10.0)  0.780 | 4.0 (4.0-6.0)  6.0 (4.0-8.0)  0.102 | 5.0 (4.0-7.0)  6.0 (4.0-10.0)  0.109 | 10.0 (4.0-14.0)  8.0 (7.3-12.8)  0.743 | 43.0 (33.0-49.5)  46.0 (37.0-50.0)  0.319 |
| Other disease  Yes  No  p* | 67  11 | 6.0 (4.0-7.0)  7.0 (6.0-9.0)  0.043 | 5.0 (4.0-7.0)  4.0 (4.0-5.0)  0.092 | 10.0 (8.0-11.0)  8.0 (8.0-10.0)  0.677 | 4.0 (4.0-5.0)  6.0 (4.0-7.0)  0.056 | 5.0 (4.0-7.0)  6.0 (4.0-7.0)  0.345 | 12.0 (8.0-14.0)  9.0 (5.0-13.0)  0.381 | 40.0 (39.0-44.0)  45.0 (34.0-51.0)  0.421 |
| Physical activity practice  Yes  No  p* | 39  39 | 7.0 (6.0-11.0)  7.0 (5.0-9.0)  0.466 | 4.0 (4.0-5.0)  4.0 (4.0-6.0)  0.699 | 8.0 (8.0-10.0)  8.0 (8.0-10.0)  0.784 | 6.0 (4.0-7.0)  4.0 (4.0-7.0)  0.279 | 6.0 (4.0-7.0)  5.0 (4.0-7.0)  0.265 | 12.0 (8.0-15.0)  8.0 (4.0-12.0)  0.069 | 46.0 (35.0-48.0)  42.0 (35.0-48.0)  0.187 |
| Treatment of hemarthrosis at the HTC  Yes  No  p* | 37  41 | 6.0 (5.0-9.0)  7.0 (5.5-10.0)  0.491 | 4.0 (4.0-6.0)  4.0 (4.0-5.0)  0.875 | 8.0 (8.0-11.0)  8.0 (8.0-10.0)  0.440 | 5.0 (4.0-6.5)  6.0 (4.0-8.0)  0.557 | 5.0 (4.0-7.0)  6.0 (4.0-8.0)  0.397 | 11.0 (5.5-14.0)  8.0 (6.0-13.0)  0.315 | 44.0 (35.0-50.0)  45.0 (33.5-50.0)  0.818 |
| Self-infusion  Yes  No  p* | 61  17 | 7.0 (5.0-9.0)  6.0 (6.0-9.0)  0.722 | 4.0 (4.0-5.0)  4.0 (4.0-6.0)  0.563 | 8.0 (8.0-10.0)  8.0 (8.0-10.5)  0.764 | 5.0 (4.0-7.0)  4.0 (4.0-6.5)  0.510 | 5.0 (4.0-7.0)  5.0 (4.0-7.0)  0.430 | 9.0 (6.5-13.5)  9.0 (4.5-13.5)  0.769 | 44.0 (35.5-50.5)  44.0 (33.5-49.5)  0.712 |
| Infusion training  Yes  No  p* | 40  38 | 6.0 (4.0-9.0)  7.0 (6.0-11.0)  0.056 | 4.0 (4.0-5.8)  4.0 (4.0-5.3)  0.483 | 8.0 (8.0-10.0)  9.0 (8.0-10.3)  0.270 | 4.5 (4.0-7.0)  5.0 (4.0-8.0)  0.446 | 5.0 (4.0-7.0)  6.0 (4.0-8.0)  0.254 | 8.0 (4.0-13.0)  10.0 (8.0-14.3)  0.265 | 44.0 (32.3-48.0)  44.5 (36.8-51.3)  0.217 |
| Duration of prophylaxis  *r*  p^†^ | 78 | 0.141  0.218 | 0.021  0.855 | 0.091  0.430 | 0.214  0.060 | 0.251  0.027 | 0.326  0.004 | 0.317  0.005 |
| Dose  ≤ 30 IU/kg  > 30 IU/kg  p* | 50  28 | 6.0 (5.0-9.0)  8.0 (6.0-11.0)  0.116 | 4.0 (4.0-5.3)  4.0 (4.0-5.8)  0.389 | 8.0 (8.0-10.0)  8.0 (8.0-10.8)  0.676 | 6.0 (4.0-7.3)  4.0 (4.0-6.0)  0.203 | 5.5 (4.0-7.0)  5.0 (4.0-8.0)  0.769 | 9.0 (6.8-15.0)  9.0 (4.3-12.0)  0.350 | 44.0 (35.8-49.3)  44.5 (33.0-52.8)  0.835 |
| Infusion frequency  2x/week  > 2x/week  p* | 10  68 | 9.5 (6.0-13.0)  6.0 (5.0-9.0)  0.035 | 5.0 (4.0-6.3)  4.0 (4.0-5.0)  0.100 | 10.0 (8.0-11.0)  8.0 (8.0-10.0)  0.204 | 6.5 (4.0-12.0)  5.0 (4.0-7.0)  0.152 | 7.5 (5.8-11.0)  5.0 (4.0-7.0)  0.006 | 10.0 (7.8-13.5)  9.0 (4.3-13.8)  0.398 | 53.5 (46.5-59.3)  43.5 (34.3-48.0)  0.003 |
| Infusion diary  Yes  No  p* | 4  74 | 4.0 (4.0-5.5)  7.0 (6.0-9.0)  0.012 | 4.0 (4.0-4.0)  4.0 (4.0-6.0)  0.274 | 6.0 (4.0-8.0)  8.0 (8.0-10.0)  0.018 | 4.0 (4.0-4.0)  5.5 (4.0-7.0)  0.057 | 4.0 (4.0-4.0)  6.0 (4.0-7.0)  0.017 | 5.5 (4.0-7.8)  9.5 (6.8-14.0)  0.042 | 28.0 (24.0-32.8)  45.0 (36.0-50.3)  0.001 |
| General health  *r*  p^†^ | 78 | 0.095  0.408 | -0.252  0.026 | 0.000  0.997 | 0.004  0.971 | 0.016  0.891 | -0.018  0.874 | -0.017  0.880 |
| Social functioning  *r*  p^†^ | 78 | -0.042  0.717 | -0.297  0.008 | -0.203  0.074 | 0.009  0.940 | -0.105  0.360 | -0.038  0.740 | -0.145  0.204 |
| Mental health  *r*  p^†^ | 78 | -0.052  0.653 | -0.260  0.201 | -0.285  0.011 | -0.197  0.084 | -0.195  0.087 | -0.210  0.065 | -0.304  0.007 |
| Vitality  *r*  p^†^ | 78 | -0.090  0.433 | -0.395  <0.001 | -0.193  0.090 | -0.138  0.229 | -0.147  0.198 | -0.200  0.080 | -0.287  0.011 |
| Bodily pain  *r*  p^†^ | 78 | 0.007  0.950 | -0.286  0.011 | -0.070  0.545 | 0.018  0.876 | 0.011  0.923 | 0.048  0.674 | -0.031  0.786 |
| Physical functioning  *r*  p^†^ | 78 | 0.119  0.300 | -0.114  0.321 | -0.012  0.915 | 0.152  0.184 | 0.199  0.081 | 0.214  0.060 | 0.190  0.095 |
| Role limitations (physical)  *r*  p^†^ | 78 | 0.128  0.264 | -0.185  0.105 | -0.127  0.267 | 0.086  0.455 | 0.125  0.275 | 0.147  0.200 | 0.093  0.416 |
| Role limitations (emotional)  *r*  p^†^ | 78 | -0.097  0.398 | -0.072  0.529 | -0.074  0.522 | -0.207  0.069 | -0.203  0.074 | 0.019  0.868 | -0.142  0.214 |
| Total SF- 36  *r*  p^†^ | 78 | 0.024  0.836 | -0.302  0.007 | -0.159  0.164 | -0.037  0.747 | -0.034  0.765 | 0.042  0.714 | -0.075  0.515 |

HTC: Hemophilia Treatment Center; IQR: interquartile range; VERITAS-Pro: Validated Hemophilia Regimen Treatment Adherence Scale-Prophylaxis.

*Mann-Whitney’s *U* test. †Pearson’s *r* correlation.

**Supplementary Table 3- Adherence to clotting factor prophylaxis (VERITAS-Pro domains) according to population characteristics**

| **Characteristic** | **Time** | | | **Dose** | | | **Plan** | | | **Remember** | | | **Skip** | | | **Communicate** | | |
| --- | --- | --- | --- | --- | --- | --- | --- | --- | --- | --- | --- | --- | --- | --- | --- | --- | --- | --- |
|  | **Adherent (n = 63)** | **Nonadherent (n = 15)** | **p** | **Adherent (n = 66)** | **Nonadherent (n = 12)** | **p** | **Adherent (n = 43)** | **Nonadherent (n = 35)** | **p** | **Adherent (n = 73)** | **Nonadherent (n = 5)** | **p** | **Adherent (n = 71)** | **Nonadherent (n = 7)** | **p** | **Adherent (n = 41)** | **Nonadherent (n = 37)** | **p** |
| Age, in years, as median (IQR) | 30.0 (20.0-40.0) | 26.0 (21.0-35.0) | 0.819† | 29.0 (19.0-37.0) | 33.0 (23.0-49.0) | 0.147 † | 31.0 (21.0-42.0) | 26.0 (19.0-36.0) | 0.212† | 30.0 (21.0-40.0) | 21.0 (17.0-31.0) | 0.177† | 30.0 (20.0-38.0) | 30.0 (22.0-40.0) | 0.151† | 30.0 (18.0-38.0) | 30.0 (22.0-40.0) | 0.606† |
| Living in Fortaleza, in n (%) | 32 (51%) | 7 (47%) | 1.000* | 35 (53%) | 4 (33%) | 0.347* | 22 (51%) | 17 (49%) | 1.000* | 38 (52%) | 1 (20%) | 0.358* | 36 (51%) | 3 (43%) | 1.000* | 20 (49%) | 19 (51%) | 1.000* |
| White, in n (%) | 10 (16%) | 7 (47%) | 0.016* | 15 (23%) | 2 (17%) | 1.000* | 9 (21%) | 8 (23%) | 1.000* | 16 (22%) | 1 (20%) | 1.000* | 14 (20%) | 3 (43%) | 0.171* | 8 (20%) | 9 (23%) | 0.784* |
| Single, in n (%) | 39 (62%) | 10 (67%) | 1.000* | 43 (65%) | 6 (50%) | 0.346* | 25 (58%) | 24 (69%) | 0.359* | 45 (62%) | 4 (80%) | 0.646* | 43 (61%) | 6 (86%) | 0.248* | 27 (66%) | 22 (60%) | 0.642* |
| Hemophilia A, in n (%) | 58 (92%) | 9 (60%) | 0.005* | 57 (86%) | 10 (83%) | 0.675* | 38 (88%) | 29 (83%) | 0.529* | 66 (90%) | 1 (20%) | 0.001* | 64 (90%) | 3 (43%) | 0.006* | 36 (88%) | 31 (83%) | 0.748* |
| Severe hemophilia, in n (%) | 58 (92%) | 12 (80%) | 0.177* | 58 (88%) | 12 (100%) | 0.346* | 39 (91%) | 31 (89%) | 1.000* | 66 (90%) | 4 (80%) | 0.427* | 63 (89%) | 7 (100%) | 1.000* | 35 (85%) | 35 (95%) | 0.268* |
| Joint disease, in n (%) | 25 (40%) | 7 (47%) | 0.771* | 30 (46%) | 2 (17%) | 0.108* | 16 (37%) | 16 (46%) | 0.494* | 29 (40%) | 3 (60%) | 0.396* | 26 (37%) | 6 (86%) | 0.017* | 18 (44%) | 14 (38%) | 0.649* |
| Other disease, in n (%) | 15 (24%) | 1 (7%) | 0.175* | 13 (20%) | 3 (25%) | 0.703* | 10 (23%) | 6 (17%) | 0.581* | 16 (22%) | 0 (0%) | 0.577* | 16 (23%) | 0 (0%) | 0.334* | 7 (17%) | 9 (24%) | 0.576* |
| Physical activity practice, in n (%) | 29 (46%) | 10 (67%) | 0.250* | 34 (52%) | 5 (42%) | 0.755* | 21 (49%) | 18 (51%) | 1.000* | 36 (49%) | 3 (60%) | 1.000* | 35 (50%) | 4 (57%) | 1.000* | 15 (37%) | 24 (65%) | 0.023* |
| Treatment of hemarthroses at the HTC, in n (%) | 32 (51%) | 5 (33%) | 0.262* | 30 (46%) | 7 (59%) | 0.534* | 20 (47%) | 17 (49%) | 1.000* | 35 (48%) | 2 (40%) | 1.000* | 36 (51%) | 1 (14%) | 0.111* | 16 (39%) | 21 (57%) | 0.173* |
| Self-infusion, in n (%) | 49 (78%) | 12 (80%) | 1.000* | 51 (77%) | 10 (83%) | 1.000* | 32 (74%) | 29 (83%) | 0.420* | 57 (78%) | 4 (80%) | 1.000* | 55 (78%) | 6 (86%) | 1.000* | 31 (76%) | 30 (81%) | 0.595* |
| Infusion training, in n (%) | 35 (56%) | 5 (33%) | 0.156* | 32 (49%) | 8 (67%) | 0.349* | 25 (58%) | 15 (43%) | 0.255* | 39 (53%) | 1 (20%) | 0.195* | 37 (52%) | 3 (43%) | 0.708* | 23 (56%) | 17 (46%) | 0.497* |
| Duration of prophylaxis, in years, in median (IQR) | 9.0 (7.0-10.0) | 9.0 (7.0-10.0) | 0.477† | 9.0 (7.0-10.0) | 8.0 (7.0-10.0) | 0.484† | 9.0 (6.0-10.0) | 9.0 (7.0-10.0) | 0.741† | 9.0 (7.0- 10.0) | 11.0 (8.0-12.0) | 0.114† | 8.0 (6.0-10.0) | 9.0 (8.0-10.0) | 0.074† | 8.0 (6.0-10.0) | 9.0 (8.0-10.0) | 0.030† |
| Dose ≤ 30IU/Kg, in n (%) | 44 (70%) | 6 (40%) | 0.039* | 43 (65%) | 7 (58%) | 0.747* | 28 (65%) | 22 (63%) | 1.000* | 48 (66%) | 2 (40%) | 0.344* | 48 (68%) | 2 (29%) | 0.091* | 26 (63%) | 24 (65%) | 1.000* |
| Frequency >2x/week, in n (%) | 58 (92%) | 10 (67%) | 0.019* | 58 (88%) | 10 (83%) | 0.647* | 39 (91%) | 29 (83%) | 0.330* | 67 (92%) | 1 (20%) | 0.001* | 65 (92%) | 3 (43%) | 0.004* | 36 (88%) | 32 (87%) | 1.000* |
| Infusion diary | 4 (6%) | 0 (0%) | 1.000* | 4 (6%) | 0 (0%) | 1.000* | 4 (9%) | 0 (0%) | 0.123* | 4 (5%) | 0 (0%) | 1.000* | 4 (6%) | 0 (0%) | 1.000* | 4 (10%) | 0 (0%) | 0.117* |
| General health, in median (IQR) | 62.0 (42.0-72.0) | 67.0 (52.0-77.0) | 0.291† | 62.0 (50.8-72.0) | 42.0 (34.5-60.3) | 0.028† | 62.0 (42.0-72.0) | 62.0 (42.0-72.0) | 0.614† | 62.0 (44.5-72.0) | 62.0 (34.5-88.5) | 0.782† | 62.0 (42.0-72.0) | 72.0 (57.0-80.0) | 0.141† | 57.0 (42.0-72.0) | 62.0 (44.5-76.0) | 0.361† |
| Social functioning, in median (IQR) | 75.0 (50.0-100.0) | 75.0 (62.5-100.0) | 0.776† | 75.0 (62.5-100.0) | 56.3 (50.0-71.9) | 0.025† | 75.0 (62.5-100.0) | 62.5 (50.0-87.5) | 0.101† | 75.0 (56.3-100.0) | 75.0 (56.3-93.8) | 0.937† | 75.0 (62.5-100.0) | 75.0 (50.0-100.0) | 0.993† | 75.0 (50.0-100.0) | 75.0 (62.5-87.5) | 0.842† |
| Mental health, in median (IQR) | 92.0 (76.0-100.0) | 88.0 (72.0-96.0) | 0.419† | 92.0 (76.0-100.0) | 80.0 (60.0-92.0) | 0.066† | 96.0 (84.0-100.0) | 80.0 (72.0-96.0) | 0.027† | 92.0 (76.0-100.0) | 72.0 (66.0-82.0) | 0.051† | 92.0 (76.0-100.0) | 92.0 (60.0-92.0) | 0.354† | 92.0 (74.0-100.0) | 92.0 (72.0-96.0) | 0.328† |
| Vitality, in median (IQR) | 70.0 (55.0-80.0) | 75.0 (55.0-90.0) | 0.615† | 75.0 (65.0-85.0) | 55.0 (51.3-71.3) | 0.003† | 75.0 (65.0-85.0) | 65.0 (55.0-75.0) | 0.018† | 75.0 (57.5-85.0) | 55.0 (50.0-77.5) | 0.229† | 75.0 (55.0-85.0) | 75.0 (50.0-95.0) | 0.951† | 75.0 (60.0-85.0) | 70.0 (55.0-80.0) | 0.381† |
| Bodily pain, in median (IQR) | 52.0 (41.0-74.0) | 84.0 (41.0-90.0) | 0.078† | 62.0 (41.0-90.0) | 41.0 (30.3-58.3) | 0.017† | 62.0 (41.0-90.0) | 52.0 (31.0-84.0) | 0.374† | 62.0 (41.0-87.0) | 51.0 (15.0-90.0) | 0.677† | 62.0 (41.0-90.0) | 84.0 (41.0-90.0) | 0.608† | 51.0 (36.0-90.0) | 62.0 (45.5-87.0) | 0.537† |
| Physical functioning, in median (IQR) | 70.0 (40.0-95.0) | 85.0 (55.0-100.0) | 0.205† | 82.5 (40.0-95.0) | 55.0 (47.5-60.0) | 0.097† | 65.0 (40.0-95.0) | 85.0 (45.0-95.0) | 0.382† | 75.0 (40.0-95.0) | 70.0 (42.5-100.0) | 0.663† | 70.0 (40.0-95.0) | 95.0 (65.0-100.0) | 0.083† | 60.0 (37.5-95.0) | 80.0 (55.0-95.0) | 0.142† |
| Role limitations (physical), in median (IQR) | 100.0 (25.0-100.0) | 100.0 (50.0-100.0) | 0.532† | 100.0 (25.0-100.0) | 12.5 (0.0-100.0) | 0.054† | 100.0 (25.0-100.0) | 50.0 (0.0-100.0) | 0.201† | 100.0 (25.0-100.0) | 75.0 (0.0-100.0) | 0.592† | 100.0 (25.0-100.0) | 100.0 (25.0-100.0) | 0.650† | 75.0 (12.5-100.0) | 100.0 (25.0-100.0) | 0.193† |
| Role limitations (emotional), in median (IQR) | 100.0 (33.0-100.0) | 100.0 (33.0-100.0) | 0.966† | 100.0 (33.0-100.0) | 100.0 (0.0-100.0) | 0.734† | 100.0 (25.0-100.0) | 100.0 (0.0-100.0) | 0.134† | 100.0 (33.0-100.0) | 0.0 (0.0-66.5) | 0.051† | 100.0 (33.0-100.0) | 33.0 (0.0-100.0) | 0.097† | 100.0 (33.0-100.0) | 100.0 (16.5-100.0) | 0.973† |
| Total SF-36, in median (IQR) | 69.1 (54.9-83.3) | 74.9 (65.6-90.5) | 0.313† | 73.2 (61.7-84.4) | 60.2 (40.5-66.4) | 0.005† | 72.8 (58.1-86.8) | 68.5 (51.7-75.9) | 0.111† | 70.3 (57.8-83.3) | 70.4 (33.8-80.1) | 0.552† | 69.6 (57.6-83.3) | 75.4 (51.7-90.5) | 0.643† | 67.4 (55.5-84.6) | 72.8 (61.0-83.2) | 0.432† |

HTC: Hemophilia Treatment Center; IQR: interquartile range; SF-36: Short Form 36; VERITAS-Pro: Validated Hemophilia Regimen Treatment Adherence Scale-Prophylaxis.

*Fisher’s exact test. †Mann-Whitney’s *U* test.

**STROBE Statement—Checklist of items that should be included in reports of *cross-sectional studies***

|  | **#** | **Recommendation** | **Page** |
| --- | --- | --- | --- |
| **Title and abstract** | 1 | (*a*) Indicate the study’s design with a commonly used term in the title or the abstract | Title page 1 |
|  |  | (*b*) Provide in the abstract an informative and balanced summary of what was done and what was found | Abstract 1 |
| **Introduction** | | | |
| Background/rationale | 2 | Explain the scientific background and rationale for the investigation being reported | 5-6 |
| Objectives | 3 | State specific objectives, including any prespecified hypotheses | 6 |
| **Methods** | | | |
| Study design | 4 | Present key elements of study design early in the paper | 6 |
| Setting | 5 | Describe the setting, locations, and relevant dates, including periods of recruitment, exposure, follow-up, and data collection | 6 |
| Participants | 6 | (*a*) Give the eligibility criteria, and the sources and methods of selection of participants | 6-7 |
| Variables | 7 | Clearly define all outcomes, exposures, predictors, potential confounders, and effect modifiers. Give diagnostic criteria, if applicable | 7-8 |
| Data sources/ measurement | 8* | For each variable of interest, give sources of data and details of methods of assessment (measurement). Describe comparability of assessment methods if there is more than one group | 7-9 |
| Bias | 9 | Describe any efforts to address potential sources of bias | 6-8 and 11 |
| Study size | 10 | Explain how the study size was arrived at | 6 |
| Quantitative variables | 11 | Explain how quantitative variables were handled in the analyses. If applicable, describe which groupings were chosen and why | 6-8 |
| Statistical methods | 12 | (*a*) Describe all statistical methods, including those used to control for confounding | 8-9 |
|  |  | (*b*) Describe any methods used to examine subgroups and interactions | 6-9 |
|  |  | (*c*) Explain how missing data were addressed | 8-9 |
|  |  | (*d*) If applicable, describe analytical methods taking account of sampling strategy | 6 |
|  |  | (*e*) Describe any sensitivity analyses | - |
| **Results** | | | |
| Participants | 13* | (a) Report numbers of individuals at each stage of study—eg numbers potentially eligible, examined for eligibility, confirmed eligible, included in the study, completing follow-up, and analysed | 9 and Figure 1 |
|  |  | (b) Give reasons for non-participation at each stage | Figure 1 |
|  |  | (c) Consider use of a flow diagram | Figure 1 |
| Descriptive data | 14* | (a) Give characteristics of study participants (eg demographic, clinical, social) and information on exposures and potential confounders | 8-9 |
|  |  | (b) Indicate number of participants with missing data for each variable of interest | 8 |
| Outcome data | 15* | Report numbers of outcome events or summary measures | 9-10 and Supplementary material 2 |
| Main results | 16 | (*a*) Give unadjusted estimates and, if applicable, confounder-adjusted estimates and their precision (eg, 95% confidence interval). Make clear which confounders were adjusted for and why they were included | 9-10 |
|  |  | (*b*) Report category boundaries when continuous variables were categorized | 8 |
|  |  | (*c*) If relevant, consider translating estimates of relative risk into absolute risk for a meaningful time period | - |
| Other analyses | 17 | Report other analyses done—eg analyses of subgroups and interactions, and sensitivity analyses | Supplementary material 4-7 |
| **Discussion** | | | |
| Key results | 18 | Summarise key results with reference to study objectives | 10-12 |
| Limitations | 19 | Discuss limitations of the study, taking into account sources of potential bias or imprecision. Discuss both direction and magnitude of any potential bias | 12-13 |
| Interpretation | 20 | Give a cautious overall interpretation of results considering objectives, limitations, multiplicity of analyses, results from similar studies, and other relevant evidence | 9-11 |
| Generalisability | 21 | Discuss the generalisability (external validity) of the study results | 12 |
| **Other information** | | | |
| Funding | 22 | Give the source of funding and the role of the funders for the present study and, if applicable, for the original study on which the present article is based | Title page 3 |

*Give information separately for exposed and unexposed groups.

**Note:** An Explanation and Elaboration article discusses each checklist item and gives methodological background and published examples of transparent reporting. The STROBE checklist is best used in conjunction with this article (freely available on the Web sites of PLoS Medicine at http://www.plosmedicine.org/, Annals of Internal Medicine at http://www.annals.org/, and Epidemiology at http://www.epidem.com/). Information on the STROBE Initiative is available at www.strobe-statement.org.


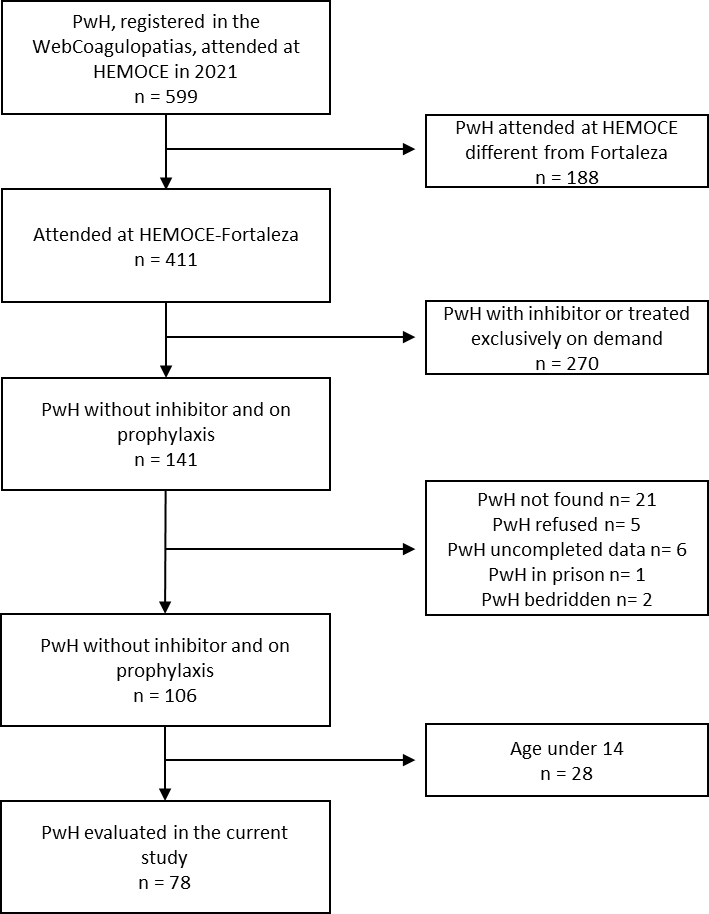


**Supplementary Figure 1- Population flowchart**

HEMOCE: Centro de Hematologia e Hemoterapia do Ceará; PwH: people with hemophilia.
